# An Energy-Efficient and High-Quality Video Transmission Architecture in Wireless Video-Based Sensor Networks

**DOI:** 10.3390/s8074529

**Published:** 2008-08-04

**Authors:** Hadi S. Aghdasi, Maghsoud Abbaspour, Mohsen Ebrahimi Moghadam, Yasaman Samei

**Affiliations:** Computer Engineering Department, Faculty of Electrical and Computer Engineering, Shahid Beheshti University, G. C., Evin, Tehran, Iran; E-mails: aghdasi@sbu.ac.ir, m_moghadam@sbu.ac.ir, ya.samei@mail.sbu.ac.ir

**Keywords:** Wireless Multimedia Sensor Network, Video Sensor Nodes, Communication Protocol Stack, Energy Efficiency, Video Quality

## Abstract

Technological progress in the fields of Micro Electro-Mechanical Systems (MEMS) and wireless communications and also the availability of CMOS cameras, microphones and small-scale array sensors, which may ubiquitously capture multimedia content from the field, have fostered the development of low-cost limited resources Wireless Video-based Sensor Networks (WVSN). With regards to the constraints of video-based sensor nodes and wireless sensor networks, a supporting video stream is not easy to implement with the present sensor network protocols. In this paper, a thorough architecture is presented for video transmission over WVSN called Energy-efficient and high-Quality Video transmission Architecture (EQV-Architecture). This architecture influences three layers of communication protocol stack and considers wireless video sensor nodes constraints like limited process and energy resources while video quality is preserved in the receiver side. Application, transport, and network layers are the layers in which the compression protocol, transport protocol, and routing protocol are proposed respectively, also a dropping scheme is presented in network layer. Simulation results over various environments with dissimilar conditions revealed the effectiveness of the architecture in improving the lifetime of the network as well as preserving the video quality.

## 1. Introduction

The advances in Micro Electro-Mechanical Systems (MEMS) and wireless communications resulted in the wireless sensor networks (WSN). These networks are comprised of large numbers of low-cost, low-power and multifunctional sensor nodes. Thus, it is predicted that wireless sensor networks will become conventional in our daily life and recently there have been a lot of hot research topics in this field of study [1]. Nowadays, a single sensor device can be equipped with audio and visual information collection modules using low-cost hardware such as CMOS cameras, array sensors and microphones. This fostered the development of Wireless Multimedia Sensor Networks (WMSN), in a way that they are able ubiquitously to obtain multimedia content such as video and audio streams, still images, and scalar sensor data from the environment [2].

Currently wireless sensor networks are used widely in multimedia streaming. Multimedia surveillance sensor networks [3], advanced health care delivery [4], automated assistance for the elderly and family monitors [5], traffic avoidance, enforcement and control systems [6], and industrial process control [7] are all instances of new WMSN applications.

Real-time multimedia streaming is used by some applications such as emergency response, video surveillance systems, battlefield, disaster discovery and indoor security, to name but a few. End-to-end delay and loss should be identified for multimedia during network transport [8]. Most of these applications use WMSN with video sensor nodes (VSN), which are called wireless video-based sensor networks (WVSN).

WVSNs were initially devised as a collection of small, inexpensive, battery operated nodes with the ability to communicate with each other wirelessly over a limited transmission range. These networks are different from traditional wireless sensor networks due to the fact that nodes are equipped with very low power cameras. These camera-nodes have the ability to capture visual information of observed areas at variable rates, process the data on-board and transmit the captured data through the multi-hop communication to the base-station (Sink) [9].

Generally, the two most important challenges for these systems are energy-efficiency and video Quality of Service. In other words, the main problem is how to simultaneously provide energy efficiency and video quality in WVSN. The huge amount of data generation and transmission by VSNs causes them to consume a lot of energy. Therefore, the limited power supply in sensor nodes becomes the bottleneck in transmitting multimedia in WVSNs [10]. On the other hand, video quality suffers from the limited power, processor, memory and radio frequency of VSNs.

Most of the previous works are devoted to image transmission [11-14] while the research on video transmission over WSN is still in the earlier stages. Therefore, in this article a new architecture for WVSN to transmit video streams, called Energy-efficient and high-Quality Video transmission Architecture (EQV-Architecture) is presented. This architecture is designed with the objectives of extending the lifetime of VSNs and increasing QoS. Thus, EQV-Architecture prolongs lifetime of VSN while preserving video quality. Literature surveys show that most of previous works considered only one of these criteria.

The application, transport, and network layers of the communication stack are customized for video transmission in EQV-Architecture to improve the performance of WVSN. In the customization procedure a new sub-application layer protocol is presented with innovative compression and prioritization algorithms. To provide suitable service for application layer it is necessary to propose new transport and network layer protocols. In the proposed transport protocol, packets are sent as bursts and inter-layer command messages are used in order that it can be aware of the status of network. Data retransmission is omitted to achieve real-time communication. Also, to improve video quality, Forward Error Correction (FEC) is suggested to be used in data link layer for high priority data. Finally, a hierarchical single-path routing protocol and a dropping scheme are invented in network layer. The proposed single-path routing protocol finds a proper path between source and Sink in the network. In order to send data bursty over a reliable path, this approach negotiates with the selected parent hop-by-hop. The dropping scheme uses probability functions for discarding packets along with considering priority level of data packets and hierarchy level of video source node. Also, EQV-Architecture can be used on Mobile Ad-hoc Network (MANET) by applying some little changes in parameters of the architecture.

The rest of this paper is organized as follows: Section 2 presents the related works on the topics of WVSN in all the related layers. The overall design of the architecture is presented in Section 3. The corresponding layers of proposed architecture, the proposed methods and protocols in the application, transport, and network layers are introduced in Sections 4, 5, and 6 respectively. Section 7 provides simulation and experimental results and finally, in Section 8 the paper's conclusions are presented.

## 2. Related Works

EQV-Architecture covers three layers of application, transport, and network. Accordingly, the most relevant work and preceding proposed protocols in each layer are introduced.

### 2.1. Application Layer Related Works

Application layer in WVSN consists of complex items with regards to WVSN specifications. QoS preservation and computation reduction in video based sensor networks are instances of these complexities. Two methods of application admission controls are presented in [15] and [16]. In [15], the aim was to increase the lifetime of the network subject to the bandwidth and reliability. In [16], admission defining depends on added energy load and application reward. Although the authors consider application level QoS, they do not simultaneously meet multiple QoS requirements (e.g. delay, reliability, and energy consumption), as required in WMSNs. Moreover, their communication optimizations need high in-network computation, which is not compatible with WVSN constraints.

Source coding is also one of application layer's services. In view of limitations of VSNs and needs of multimedia transmission, coding techniques should have high ratio of compression, low complexity and error resiliency [2]. However, traditional compression and coding like MPEG, H.263 [17] or H.264 [18] have complex encoders which do not provide energy efficiency. A group of coding is distributed source coding [19] and [20] which use a lot of energy to fuse captured images.

### 2.2. Transport Layer Related Works

The traditional transport protocols that are currently used for the Internet (i.e., UDP and TCP) cannot be directly implemented for WSNs. The TCP based protocols suffers from retransmission overhead, delays and high energy consumption [21, 22]. For real-time applications like streaming media, the User Datagram Protocol (UDP) is preferred to TCP since timeliness is more important than reliability. UDP does not provide required reliability which is needed for many sensor applications. Also, it does not offer flow and congestion control. Therefore, using it leads to packet loss and unnecessary energy consumption.

With regards to constrains of UDP, recently new proposals using multi-path transmission have been introduced such as COngestion Detection and Avoidance (CODA) [23] and Multi-flow Real-time Transport Protocol (MRTP) [24]. CODA has considerable delay since it decides on the basis of the status of the intermediate nodes and MRTP does not regard energy efficiency in WMSN [2].

Some other transport protocols have been designed recently for scalar WSNs with the aim of decreasing energy consumption, providing reliability, and controlling congestion. These protocols did not fulfill major factors of QoS, such as high bandwidth and real-time communication, which are required for multimedia communication in sensor networks, e.g. RMST [25], RBC [26], and STCP [21] do not support real-time communication while providing reliability. Also, Fusion is another transport protocol that is not compatible with the limited energy sources of video sensor nodes [27].

### 2.3. Routing Protocol Related Works

Various surveys have been conducted on multimedia transmission in sensor networks and each of them categorized routing protocols differently. According to the classification of present routing protocol in [2], three major classes of routing protocols are: network condition based, traffic classes based, and real-time streaming based. To the best of our knowledge, the most closely related works are as follows: in network condition class, imaging data are employed in routing. In [28], constructing relative geographical topology of the network for routing along with adaptive priority leads to the ability of supporting the event driven applications.

A multi-path routing protocol in [29] routs packets which are divided to two classes of best effort and real-time. Although energy consumption and QoS optimized in this protocol, it does not support multiple priorities with different QoS requirements for real-time traffic. Moreover, in order to compute paths, each node should have the entire topology of the network which makes this protocol not scalable.

The proposed protocol in [4] presents an approach for using mobile sensor networks in telemedicine applications. This protocol tries to modify the third generation mobile sensor network to achieve QoS. It assigns various priorities to generated data with regards to criteria including latency tolerance and hand-off dropping rate.

Real-time communication is the major issue in [30] and [31]. SPEED gives a definition of speed and forwards packets by calculating speed. It guarantees that packet will arrive after certain delay under this condition [30]. Any packet which requests less delay will not be admitted for transmission. SPEED regards delay as well as congestion; it uses a technique called *back-pressure re-routing* to mitigate congestion. Mentioned technique prevents forwarding packets over congested links. Although SPEED has positive points, it does not provide prioritization and also avoids sending packets with higher speed than the defined threshold. In [31], QoS differs in timeliness and reliability. In order to obtain these differences multi-path routing and multiple delivery speed are provided.

Another routing protocol which addressed both energy efficiency and quality of service is: A Link Quality Estimation based Routing for Wireless Sensor Networks (LQER) [32]. This protocol considers historical link states and link quality estimation for routing. LQER constructs a connectivity graph based on the result of link quality estimation and uses dynamic windows for capturing historical link states. The link quality estimation policy results in reliability as well as energy efficiency for LQER.

Energy aware routing protocol is explained in Battery-Aware Routing for Streaming Data Transmissions in Wireless Sensor Networks (BAR) [10]. The discrete time model of battery discharge behavior is one of the properties of BAR. Main idea of this policy is scheduling for batteries recovery alternatively for extending the lifetime of the network. Since energy is not the only crucial parameter in WMSN, BAR could not fulfill all multimedia transmission requirements.

Recently, the authors in [33] proposed an enhancement to IEEE 802.15.4 Medium Access Control (MAC) for supporting multimedia service in wireless sensor networks, named Traffic and Energy Aware IEEE 802.15.4 (TEA-15.4). It adaptively adjusts the active period based on traffic information. Whereas, TEA-15.4 protocol related to data link layer of communication protocol stack, hence more details about this protocol and other related data link layer protocols are not discussed in this literature.

## 3. Overall Design Architecture

The available protocols are not useful for video transmission on WMSN because they do not satisfy all the requirements of WMSN nor are they designed for other network architectures, e.g., ad-hoc networks. Also, available protocols in each layer do not provide proper services to proposed custom layers; therefore, a new architecture should be designed. This new architecture is called EQV-Architecture.

**Figure 1.**
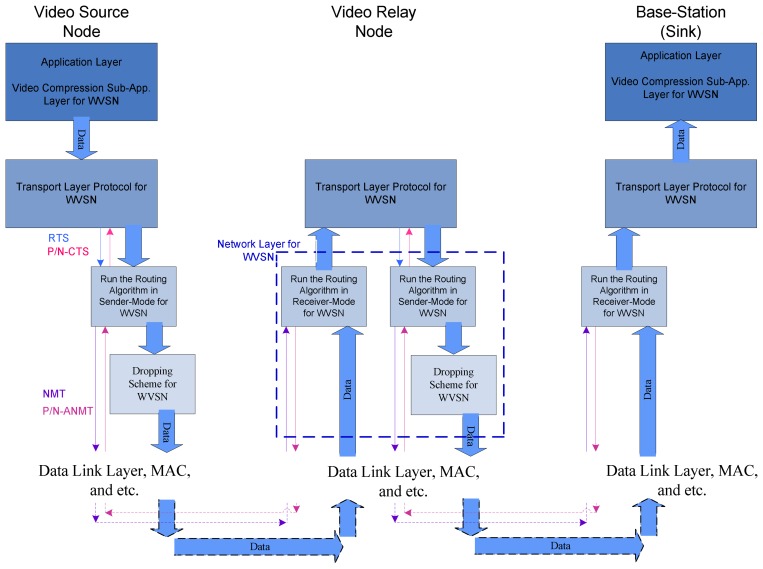
Overall EQV-Architecture

The proposed architecture provides energy efficiency and video Quality by customizing three layers of the wireless sensor networks communication protocol stack. The customized layers are application layer, transport layer, and network layer. Some new policies, algorithms, and protocols in these layers are presented. These three layers and their relations have been shown in Figure 1, and are discussed briefly in the following subsections.

### 3.1. Video Compression Sub-Application Layer Protocol for WVSN

Since video requires huge amounts of data in comparison with other kinds of information, reducing its size without interfering video quality decreases bandwidth usage and increases the communication speed. Hence, in the application layer a video compression sub-layer, which consists of a new compression model that differentiates video frames and prioritizes packets, is proposed. This compression sub-layer observes major points of video transmission as far as possible. Also, it provides data with some extra information to be used in other layers. The details of video compression sub-layer are presented in Section 4.

### 3.2. Real-Time and Reliable Transport Layer Protocol for WVSN

For transport protocol of WMSN reliability, low delay and real-time services are the aspects involved. The proposed protocol in transport layer is aimed to meet the expectation of the concerned aspects by using information which is provided by upper layer. Transport layer protocol forwards data packets without using retransmission techniques while it takes account of reliability. In addition, this protocol provides inter-layer command messages that are used in other layers to achieve real-time transmission. Section 5 explains the proposed transport layer protocol in detail.

### 3.3. Routing Protocol and Innovative Dropping Scheme in Network Layer for WVSN

Network layers play an important role in delivering the multimedia from video source node to the Sink. As a result, energy-efficiency and video quality are influenced by the approaches of this layer that include routing protocol and dropping scheme. The presented routing protocol forwards packets using a hierarchical architecture topology and an adaptive single-path transmission. In used single-path transmission protocol, energy efficiency increases more remarkably than multi-path transmission protocols which were proposed previously. In this layer, a dropping scheme that causes nodes to save energy and to prolong the lifetime of the network is proposed. On the basis of energy level of each node and information that has been provided by video compression layer inside the received packet, dropping scheme decides to discard data packets. As regards the structure of the network layer, video quality is preserved while energy consumption is distributed fairly among all the nodes. Routing protocol and new dropping scheme are described more in Section 6.

## 4. Video Compression Sub-Application Layer Protocol for WVSN

This sub-layer, located in application layer (App.), consists of a model for compressing and transmitting video in WMSN. The proposed model is based on MPEG-2 [34] and is called M-MPEG (Modified-MPEG). M-MPEG model provides facilities to overcome available constraints in VSNs such as bandwidth and energy. It is designed compatible with other layer services; therefore, the generated packets in this model consist of additional information which is used in the services of other layers such as proposed video transport and network layer. The user can adjust the energy consumption, video quality, and bandwidth usage in this sub-layer.

M-MPEG supposed that each video frame is in gray-scale. The resolution of video frames should be adapted to the need of each application. The M-MPEG utilizes two types of video frames. The first type is named Main-Frame (M-Frame), and second is named Difference-Frame (D-Frame). These types and their methods are described below.

### 4.1. M-Frame

M-Frames are compressed before transmission, using an extended JPEG method [34]. Compared with JPEG, the extended JPEG method has an additional step that prioritizes image blocks. This step is called priority level step. Details are presented in the next sub-sections.

#### 4.1.1. M-Frames Compression

**Figure 2.**
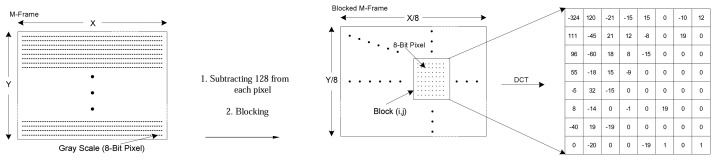

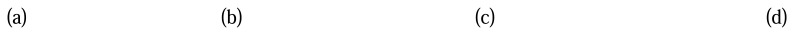
a) M-Frame Structure, b) The operations are done over M-Frame, c) M-Frame Blocked structure, and DCT is performed over Block_ij_, d) An 8×8 matrix of DCT coefficients called: *T*.

**Figure 3.**
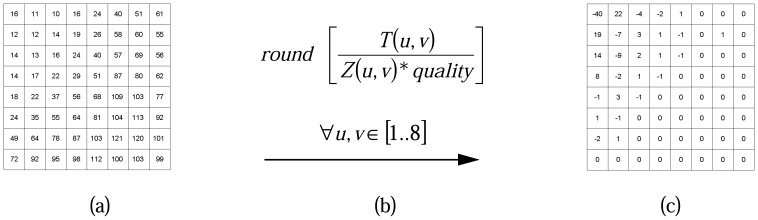
a) The standard luminance matrix called: *Z*, b) The operation is performed over both *T* and *Z* matrixes, c) An 8×8 Quantized matrix called: *Q*.

**Figure 4.**
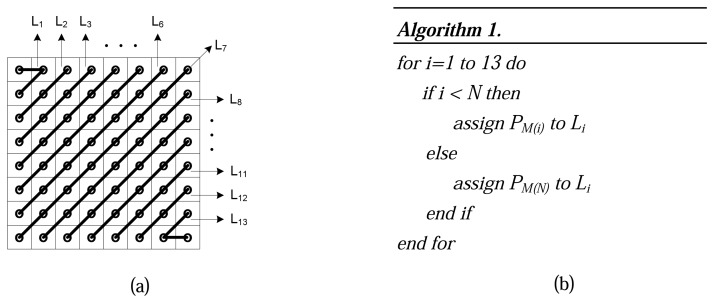
a) The used scheme for leveling, b) Algorithm to assign priority levels

In M-MPEG, M-Frame compression has been performed in 5 steps sequentially:

Step 1: *128* is subtracted from each pixel of M-Frame to put zero in the middle of the range [34]. Next, each M-Frame is divided up into 8×8 blocks without overlapping. An example is shown in Figures 2 (a, b, and c).
Step 2: DCT [35] is applied to each block independently. The output of each DCT is an 8×8 matrix of DCT coefficients as shown in Figure 2.d.
Step 3: In this phase that is called quantization phase; the less important DCT coefficients are wiped out using quantization matrix. To do this, each of the coefficients in the 8×8 DCT matrix (*T*) is divided by the corresponding standard quantization matrix elements (*Z*) [34]. The *quality coefficient* can be used to adjust compression ratio to get the expected video frame quality. Figure 3 exemplifies this step.
Step 4: The elements of each block are partitioned into *13* levels as it is shown in Figure 4.a. This structure is formed in order of importance of DCT quantized coefficients in each block. Also, this scheme facilitates linearization which happens in the next step. Each level has a priority that is assigned to it using the Algorithm.1 in Figure 4.b.
  In Figure 4.b, *N* defines the number of priority levels that are chosen for transmission. This value is selected by the user with regards to the constraints and specified application. It determines the amount of sent pixels, thus, *N* has a direct influence on video quality and energy consumption. Since Level-1 *(L_1_)* contains a DC component and two major low frequency AC components (as shown in Figure 4.a), it has a higher priority and, as is shown in Figure 4.b, *P_M(1)_* is dedicated to it. Thus, it is necessary to send the data packets with priority *P_M(1)_* to the Sink in a more reliable way. To achieve full reliability, all communication stack layers cooperate with each other. *P_M(N)_* is the lowest priority because the most of elements in this level are zero DCT coefficients. Data packets with priority level *P_M(N)_* are discarded immediately because they have not great effects on video quality. *P_M(2)_* to *P_M(N-1)_* are assigned to the other levels from *2* to *N-1*, respectively. Data packets with these priority levels are transmitted using semi-reliable policies. These policies have used different probabilities for transmitting data packets. Policies and the way in which probabilities are assigned to each level are described in Sections 6.2.
Step 5: The *64* elements of each block are linearized and run-length encoding is applied to the result. ZigZag transform is used to linearize block elements.

After end of these steps M-Frame data packets are transferred to the next communication layers where hop-by-hop transmission is used to deliver them to the base-station (Sink).

### 4.2. D-Frame

The result of subtracting current frame from M-Frame is called D-Frame. M-Frame in both receiver and transmitter is buffered until the next M-frame is transmitted. Therefore, by using a D-Frame we can construct a new video frame drawing on the buffered M-Frame at receiver end.

#### 4.2.1. D-Frames Compression

**Figure 5.**
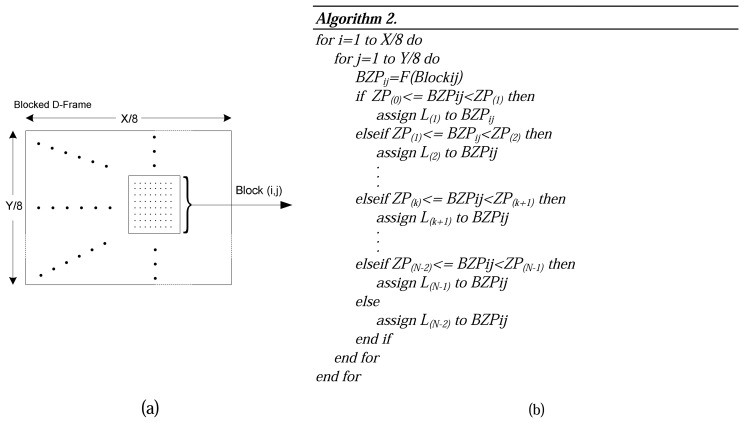
a) Blocked D-Frame, b) Algorithm to assign levels to the D-Frame blocks.

The operations performed to transmit the D-Frame are completely different from MPEG-2. D-frame compression and transmission method is described in the following steps:

Step 1: D-Frame is generated as explained above. Each D-Frame is divided into 8×8 blocks.
Step 2: The number of zero elements of each block_ij_ (*BZP_ij_*) is computed by using function *F*(*Block_ij_*). The maximum number of *BZP* is *64*, which is divided into *N* equal parts with *ZP_(k)_* and *ZP_(k+1)_* as boundaries. A level is assigned to each block of Figure 5.a, using Algorithm.2 presented in Figure 5.b.
  Then a priority is assigned to each leveled block using following relation:
  (1) $$\forall \text{Blockij}\;\left(1<=i<=X/8\;\text{and}\;1<=j<=Y/8\right)\;\text{gets}\;P_{D(t)}\;(1<=t<=N)\;\text{iff}\;L_{\left(t\right)}\;\text{be assigned to}\;\text{Blockij}$$
  D-Frame packets with priority level (*1*) (*P_D(1)_*) are very important and have the maximum number of dissimilar pixels. They should be delivered to the base-station (Sink). Therefore, M-MPEG uses highly reliable schemes to transmit this type of packets (More details are presented in next sections).
  It is not necessary to transmit the packets with priority *P_D(N)_* because these types of packets have minimum number of dissimilar pixels and their transmission does not important effect in video quality. The *P_D(2)_* to *P_D(N-1)_* are delivered to the Sink by using semi-reliable policies. In these policies, each source or relay node in communication transmits the packets to the next hop employing their probabilities. Probability assignment is described in Section 6.2.
Step 3: In this step, run-length coding and then Huffman coding are applied to packets. Finally, packets with different priority levels are transmitted to lower layers of communication protocol stack.

### 4.3. M-Frame Transmission in Dynamic Periods

MPEG-2 transfers I-Frames at regular periods. Thus, it requires extra bandwidth and has high energy consumption. M-MPEG model uses the adapted period to transmit M-Frame because in the WVSNs applications camera and background are often stationary and no fast moving object exists. This idea leads to many constraints of VSNs to be observed on the part of the M-MPEG. These adapted periods are selected in accordance with variations of background that is described in the following.

The proposed adapted M-Frame transmission is based on the number of pixels that should be transmitted in M-Frame and D-frame packets. Therefore, two functions were defined to determine when an M-Frame should be transmitted instead of a D-Frame. These functions are called SPMF (Sending Pixels in M-Frame method) and SPDF (Sending Pixels in D-Frame method).

#### 4.3.1. SPMF

This function is employed to find total number of pixels, which will be sent, with applying M-Frame compression method. It is defined as follows:

(2) $$\text{SPMF}=\left(\frac{X\times Y}{64}\right)\times \sum_{i=1}^{N-1}\left(PP_{M(i)}\right)\times (1-P_{i})$$

In this equation, *X* and *Y* are video frame resolution in the columns and rows of video frame respectively. *PP_M(i)_* is pixel numbers in priority level (*i*) of each M-Frame block, and *P_i_* is probability of dropping data packets with priority level (*i*).

#### 4.3.2. SPDF

The function is used to find total number of pixels which will be sent using D-Frame compression method. It is defined as follows:

(3) $$\text{SPDF}=\sum_{i=1}^{N-1}(BP_{D(i)})\times (1-P_{i})\times AP_{i}$$

**Figure 6.**
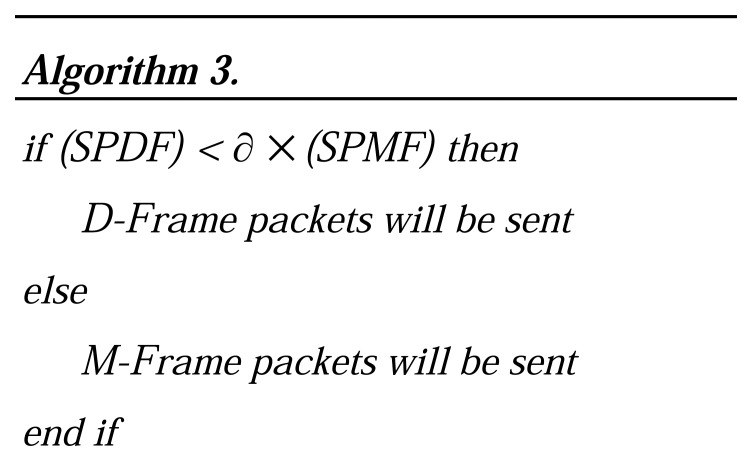
Algorithm to decide for transmitting M-Frames

In this equation, *BP_D(i)_* is number of blocks with priority level (*i*), *AP_i_* is the average of priority level range and *P*_i_ is probability of dropping data packets with priority level (*i*). The Algorithm.3 in Figure 6, is used to decide when an M-Frame should be transmitted.

In this algorithm, ∂ ∈ [0, 1] is a coefficient that affects energy saving and video quality. Using a lower value of ∂ leads to M-Frame being sent to a high frequency, which results in more energy and bandwidth consumption. It causes, however, better video quality. Higher values of ∂ causes lower video quality and more energy and bandwidth saving.

## 5. Real-Time and Reliable Transport Layer Protocol for WVSN

In this article, a new transport layer protocol is designed to satisfy constraints of video sensor nodes and required QoS in video transmission. To support data packets that are generated by video compression sub-layer, priority-based-transport services are provided. New transport layer protocol which is called real-time and reliable video transmission transport layer protocol utilizes upper layer information to provide these services.

To achieve real-time video transmission, proposed video transport protocol does not retransmit data packets. Instead some methods are employed to satisfy the reliability as a substitution. The M-Frame and the first priority level of D-Frames as mentioned in Section 4.2 are the most important data in video transmission. Therefore, transport protocol endeavors frequently to establish a connection for transmitting first priority level of M-Frames. In other priority levels, M-Frame take precedence over D-Frame in the same priority levels. Moreover, it is suggested that data link layer applies reed-Solomon forward error correction [36] to M-Frames and first priority level of D-Frames. In the following, new protocol transmission operations are discussed in two phases.

### Negotiation with Network Layer phase

In this phase, video data frames have been received from sub-application layer (in the case that current node is the capture video node) or network layer (in the case that current node is a relay node). Then, received video frames are extracted in order of priority. For delivering each priority level in a reliable way, three inter-layer command messages are used between transport and network layers: *Request To Send* (RTS) and *Positive/ Negative Clear To Send* (P/NCTS).

RTS is used to notify the network layer that there is data with selected priority level of a frame to be sent. RTS contains the type of the frame and priority level of the data. Connection is established separately for each priority level of frames. Before forwarding the whole data of the priority level, an RTS must be sent to every priority level to achieve real-time video transmission.

The RTS response is CTS that network layer forwards to transport layer. The CTS command message contains a positive or negative flag which notifies transport layer of the ability of the network layer to transmit data. The PCTS means that a valid path exists for the selected priority level and NCTS shows the opposite.

In case PCTS is received from network layer, data transmission continues with *Data transmission phase*. Otherwise, type of frame and priority level defines next step as following:

- Priority level (*1*) of M-Frame: In this case, if the Number of Retransmissions (*NoR*) is less than a threshold, another RTS is retransmitted for current priority level to the network layer. Otherwise if *NoR* passes the threshold; VSN goes to sleep-mode.
- Other priority levels of M-Frame: Whole data of current priority level is discarded, and if any other priority level exists in this frame, another RTS is sent for next priority level. In the case that, no other priority level exists, RTS is sent for the first priority level of the next frame. In other cases transmission continues in accordance with the received CTS.
- D-Frame: Regardless of priority level, whenever NCTS is received for priority level of D-Frame the entire frame is dropped and negotiation is started for next video frame.

#### Data Transmission phase

By receiving the PCTS, network layer shows that it finds proper parent for forwarding the specified priority level. Therefore entire data of selected priority level is transmitted bursty through network layer. After data transmission is completed, negotiation is started for next priority level. If sent priority level is the last priority level of the current frame, transmission proceeds to *Negotiation with Network layer phase* for next video frame.

**Figure 7.**
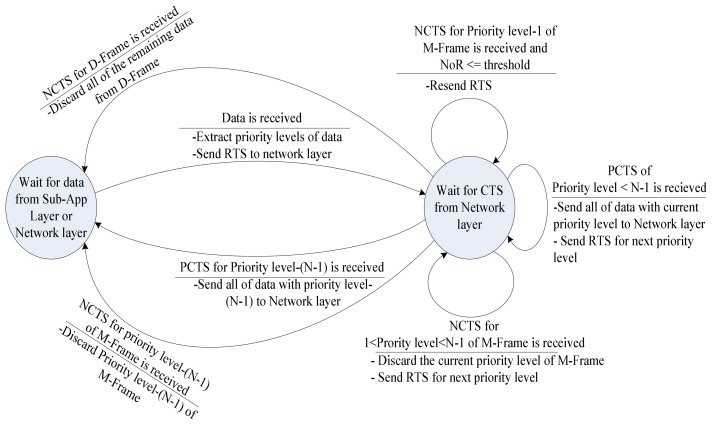
Transport protocol state-machine. The edge caption is interpreted as: events are above each line and operations are under the lines.

For implementing the proposed transport protocol, a simple state-machine is suggested. In order to describe the operations of the transport protocol two waiting states are presented in the state-machine. The conditions of switching between states beside tasks of each transport protocol phase are mentioned on the edges as shown in Figure 7.

As regards the fact that RTS can be sent to first priority level of M-Frames several times, if it exceeds the threshold –as was mentioned above– transport layer goes to sleep-mode and notifies networks layer in receiver-mode (relay node) or sub-application layer (source node). Also, when the network layer sender assures that no parent is available for transmission, regardless of the number of RTS retransmission, it forces transport layer to be in sleep-mode. Transport layer stays in sleep-mode until hierarchical network topology is reconfigured (see Section 6.1).

## 6. Network Layer for WVSN

The proposed network layer provides two services: *single-path routing protocol* and *dropping scheme*. Proposed routing protocol finds a single-path adapted with nodes status (energy and topology) and data importance for video transmission. Dropping scheme is a policy for reducing bandwidth and energy consumption of nodes without compromising the quality of services such as video quality. This scheme works by dropping low priority packets with regard to node-Sink distance. Single-path video routing is provided using inter-layer/inter-node command messages which are received from transport layers and negotiating with remote node. Dropping scheme is provided by using information embedded in data packets in compression sub-application layer and routing protocol like priority level of the data and hierarchy level of the capture node.

### 6.1. New Energy-Efficient and Single-Path Routing Protocol for WVSN

The main task of wireless sensor nodes is to sense and collect data from a target domain, process the data, and transmit the information back to the base-station (Sink) in all applications. Therefore, development of energy-efficient routing protocols is necessary to set up paths between sensor nodes and the Sink. In addition, to use this protocol in wireless video-based sensor networks, it should provide high quality video transmission.

The proposed routing protocol is a single-path routing based on hierarchical network topology. In EQV-Architecture, a Hierarchical Data Aggregation scheme for sensor networks (HDA) [37] is utilized for configuring hierarchical topology. The routing protocol supposed that in each time Sink requests to receive a specified video from the source node only, and the aim of routing is to find an energy-efficient single-path from the source node to Sink while preserving video quality. This protocol is called Energy-efficient Single-path Routing Protocol.

**Figure 8.**
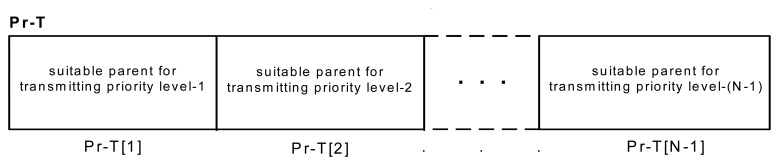
Priority Table

The proposed routing protocol consists of two kinds of tables: Priority-Table (Pr-T) and Parent-Table (Pa-T). Pr-T is an *N-1* cell array that *i*-th cell content shows the parent which can forward packets with *i*-th priority level. *N* is the maximum priority level that is defined by user in compression layer. As was mentioned in Section 4, only data with priority (*1*) to (*N-1*) will be transmitted. Every time after reconfiguring the hierarchical network structure, cells of Pr-T are set to zero. This table is shown in Figure 8 in detail.

**Figure 9.**
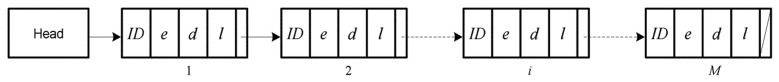
Parent Table

Pa-T is a link list data structure that includes information that a child has about status of its parents. The fields of each node of link list are: parent *ID*, energy (*e*), distance (*d*) and also the minimum priority level (*l*) that this parent can transmit as is shown in Figure 9.

When hierarchical structure of the network is reconfigured, in addition to required information for constructing hierarchical tree, each node sends some data to its children to update their Pa-T. Using this information, fields of Pa-T are filled with updated energy, distance, and minimum priority level values of parents. The priority level value that each node transmits to its children is the result of the following equation:

(4) $$\text{HighestPriority}\;\{P_{k}‐1,\;\text{LowestPriority}\{P_{1},P_{2},\ldots ,P_{i},\ldots ,P_{M}\}\}$$

Where *P_k_* -*1* is the maximum priority level that its current parent is able to transmit, and it is computed on the basis of node energy independent from situations of other nodes. The computation method of *P_k_* is explained in Section 6.2.1. *P_i_* stands for priority level that the parent *i* in Pa-T list of current parent can transmit and *M* is the number of the node's parents.

In the proposed routing protocol, Pr-T and Pa-T need more memory than does the conventional routing protocols. Unlike energy, memory is a reusable resource. Therefore, it is reasonable to use a little more memory for achieving energy efficiency.

Depending on whether the proposed routing protocol is in sender-mode or in receiver-mode of the VSN, it works differently. Two dissimilar algorithms for mentioned modes are defined below.

#### 6.1.1. Routing protocol for Sender-Mode of VSN

In order to forward the received data in transport layer to next hop in a reliable way, the proposed single-path routing protocol goes through sender-mode phases as is delineated below:

##### Parent Selection phase

This phase begins when an RTS is received from the transport layer. An appropriate parent is chosen by drawing on Pr-T and Pa-T in accordance with priority level which was mentioned in RTS. If any value other than zero exists in cell of Pr-T pointed to by priority (*l*) (Pr-T [*l*]), the value shows the relevant parent *ID*. Node selects this parent as a node to communicate with. In a case that Pr-T [*l*] content is zero, algorithm refers to Pa-T. Considering the parents which Pa-T[*l*] has the lowest priority level, the most appropriate parent according to energy and distance is selected by computing the value of *f(e,d)*. Therefore, the parent with maximum value of *f(e,d)* is chosen and the *ID* of the chosen parent will be put in all zero cells of Pr-T and *Remote Negotiation phase* begins. If no acceptable parent exists in Pa-T, NCTS is sent as a notification to the transport layer and the algorithm waits for another RTS from *Parent Selection phase*.

In order to distributing the energy consumption in the network and having fair energy management, *e* is used as the major factor for parent selection, and *d* is taken in to account to decrease hop-by-hop delay. Therefore, *f(e,d)* is defined as *e^2^/d*. Also, some other literatures deployed energy and distance as two parameters for choosing next hop node as well in similar manner [38] and [39].

##### Remote Negotiation phase

Three inter-node command messages are utilized for negotiating with remote node (selected parent) in transmission. After selecting a proper parent a message called Negotiation Message for Transmission (NMT) is forwarded to the selected parent. Next operation of routing protocol is influenced by Answer to NMT (ANMT) which could be positive (PANMT) or Negative (NANMT). Three conditions could occur at this stage:

- Receiving PANMT: In this case, a PCTS is forwarded to transport layer to notify it and routing continues from *Preparing Data for Remote Transmission phase*.
- Receiving NANMT: Both Pa-T and Pr-T are updated by receiving NANMT. Priority field of selected parent in Pa-T is updated with value of priority level field of NANMT. Pr-T also changes; all cells with the *ID* of selected parent are set to zero. Also, NCTS is sent to transport layer and routing proceeds from *Parent Selection phase*.
- Timeout: When selected parent does not respond to NMT in a specified time interval, the timer times out; then Pa-T and Pr-T become updated. Selected parent is eliminated from PaT and also all cells of Pr-T which contain the *ID* of this parent are revalued to zero. Moreover, NCTS is forwarded to transport layer and routing starts from *Parent Selection phase*.

###### Preparing Data for Remote Transmission phase

In this phase, data is received from transport layer and is put through dropping scheme (see Section 6.2.). Then, all data from current priority level is forwarded to the selected parent bursty. The routing is started from *Parent Selection phase* after sending entire data to remote parent.

**Figure 10.**
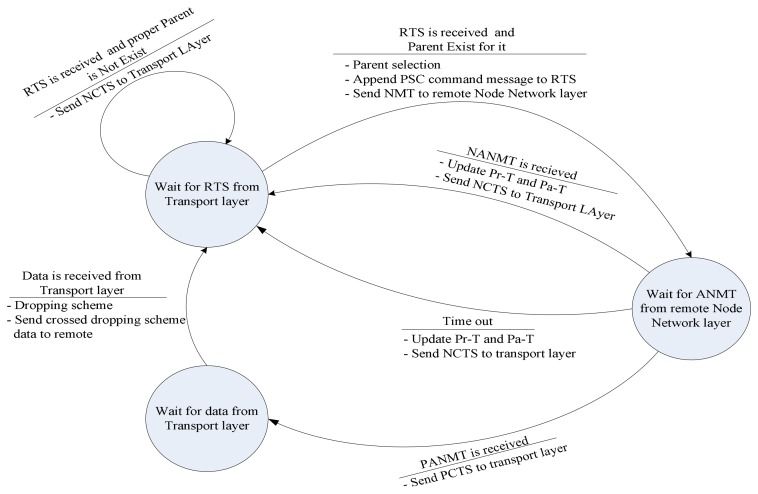
Routing protocol state-machine for VSN in sender-mode

Figure 10, shows a three-state state-machine which is used to reveal routing protocol in sender-mode. In this state-machine, conditions and operations are laid at the edges.

Assuming the condition that no parent exists in Pa-T, routing protocol in sender-mode notifies transport layer and goes to sleep-mode. Whenever hierarchical network topology is reconfigured, routing protocol changes its mode and starts from *Remote Negotiation phase*.

#### 6.1.2. Routing protocol for Receiver-Mode of VSN

##### Checking for Proper Parent phase

When NMT is received from the remote node, routing protocol responds to it either by PANMT or NANMT depending on its energy status and Pa-T. In a case that it has a suitable parent it responds by PANMT and goes to the next phase. Otherwise, the priority level field of NANMT should be filled using the result of Equation 4. Then NANMT is forwarded to remote node and routing waits for another NMT.

##### Receiving Data phase

In this phase, whole data of the same priority level is received and delivered to transport layer. The routing operation begins from *Checking for Proper Parent phase*.

**Figure 11.**
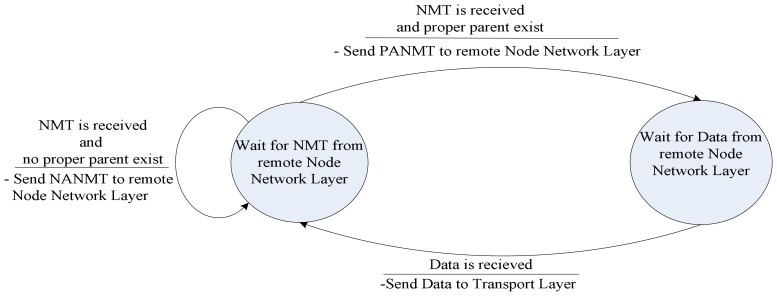
Routing protocol state-machine for VSN in receiver-mode

Routing protocol in receiver-mode also can go to sleep-mode under specified condition. When transport layer is in sleep-mode it also notifies routing protocol to go to sleep-mode and not to respond to NMT. Routing protocol in receiver-mode keeps on routing whenever hierarchical network topology is reconfigured. The operation of routing protocol in receiver-mode is clarified by the state-machine in Figure 11.

### 6.2. Dropping Scheme

Dropping process consists of two stages; at the first stage, each node decides which priority level should be sent and at the second stage each node drops some packets randomly while taking account of the priority level of the packets and distance between current node-Sink.

#### 6.2.1. Energy Aware Dropping

**Figure 12.**
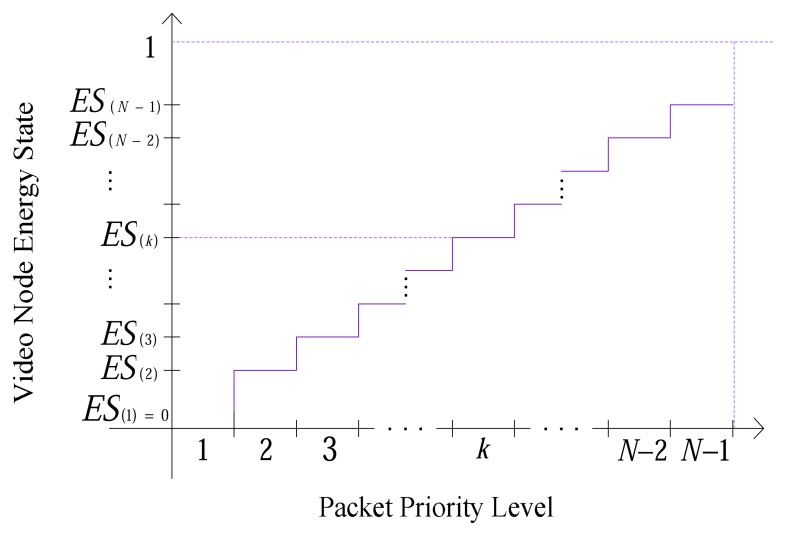
Energy aware dropping scheme based on Energy-State of node

Clearly the energy of each active video node decreases over time. Therefore, discarding priority level is calculated on the basis of normalized energy level of a node. All received packets with this priority level and lower are discarded. As shown in Figure 12, each priority levels *P_1_, P_2_, …, P_k_, …, P_N-1_*, is associated with a normalized energy level *ES_(1)_*, *ES_(2)_*, …, *ES_(k)_*, …, *ES_(N-1)_* respectively. These levels should be specified by the user regarding to the application. *P_k_* stands for both *P_M(k)_* and *P_D(k)_* as mentioned in Section 4.1.1 and 4.2.1, respectively and for each priority level (*k*) ∈ N, there exists *ES_(k)_* ∈ [0,1) such that *ES_(k)_* < *ES_(k+1)_*. For example, if energy of a node becomes less than *ES_(k)_*, then packets with priority level (*k*) and lower priority levels are not transmitted.

In order to reach a proper energy level for each priority level that is compatible with the requirement of application as well, Equation (5) is suggested:

(5) $$ES_{\left(l\right)}=\{\begin{array}{ll} {\left(\frac{l}{N}\right)}^{z} & \text{if}\;1<l<N\\ 0 & \text{if}\;l==1 \end{array}$$

**Figure 13.**
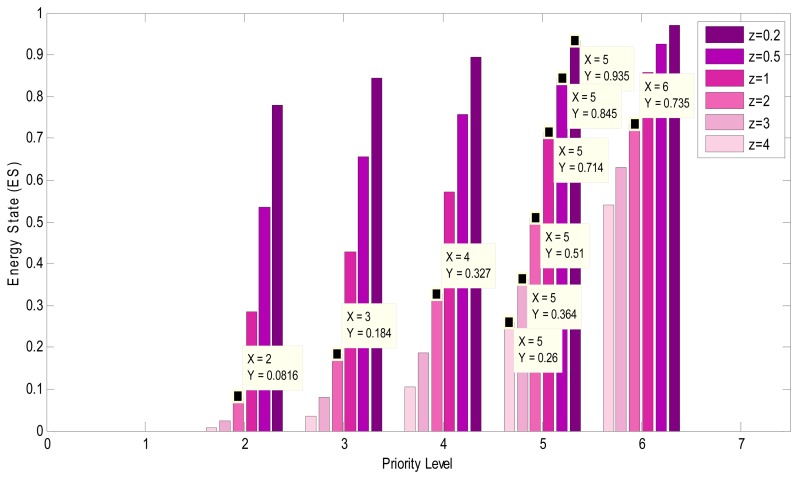
Values of *ES_(l)_* using different z

In this equation, *N* is the maximum number of priority levels and (*l*) is the current priority level. *z* is used to make Equation (5) adaptable with different applications. For instance, if in an application, lifetime of the network is the major point and normal video quality is sufficient, choosing *z* <*1* in the equation will lead to high values for *ES_(l)_*. Consequently, nodes start to drop priority levels more quickly. Therefore, video quality decreases when lifetime of network improves significantly. Conversely, for applications whose concerning point is video quality, values greater than *1* for *z* causes nodes to begin dropping in lower level of energy. In all simulations *z* is considered *2*. Figure 13, shows different *ES_(l)_* calculated for various values of *z*.

The energy-aware dropping scheme uses Equation (5) to define the priority level which can be sent. This scheme first maps the domain of the nodes energy between *0* and *1*, and then calculates the result of Equation (5) according to current priority level (*ES_(l)_*). After comparing the mapped energy with *ES_(l)_*, it will be determined whether or not this priority level can be transmitted. If mapped energy of the node is greater, node is allowed to send the data of the priority level; otherwise the node is unable to send the priority level. For example by choosing the value *2* for *z* in Figure 13, if a mapped energy of a node equals *0.4* then *ES_(4)_* is *0.327* for data packets with priority level (*4*). In this example since *ES_(4)_* is less than mapped energy, node passes them to the next stage. Unlike for priority level (*5*) which calculated *ES_(5)_* is *0.5*, the node drops packets.

If a node cannot transmit packets with specific priority level and lower than that, its child is not allowed to send packets with this priority level and lower to this node. The node notifies its children by ANMT when they ask for communication. If all parents of a node cannot send packets with specific priority level and lower, that node itself cannot send packets as well.

#### 6.2.2. Random Early Dropping

Some packets are dropped before arriving at *energy aware dropping* so that the quality of video is still satisfactory. Applying random early dropping to data packets which are not dropped in energy aware dropping also helps increase the lifetime of the network. Early dropping of the data packets occur on the basis of node energy and hierarchical level with considering the priority and influence of these packets on video quality. Packet selection for early dropping consists of two phases: *energy level* and *hierarchical level*. All packets selected in both *energy level* and *hierarchical level* selection phases are early dropped. In the following, two packet selection policies are described.

##### A. Energy level based packet selection

This selection benefits from video compression layer prioritization. The probability that a packet is dropped depends on the priority level of the packet and energy level of the node. The packet with first priority level never drops.

Dropping probabilities *P_dr(1)_*, *P_dr(2)_*, …, *P_dr(k)_*,…, *P_dr(N-1)_* are assigned to each priority level *P_1_*, *P_2_*, …, *P_k_*, …, *P_N-1_* respectively. Where *P_k_* stands for both *P_M(k)_* and *P_D(k)_* and for all (*k*) ∈ N, *P_dr(1)_*=*0*, *P_dr(k)_* ∈ [0,1]. Since the content of packets with priority level (*k*) is most important than content of packets with priority level (*k*+*1*) then *P_dr(k)_*<*P_dr(k+1)_*.

**Figure 14.**
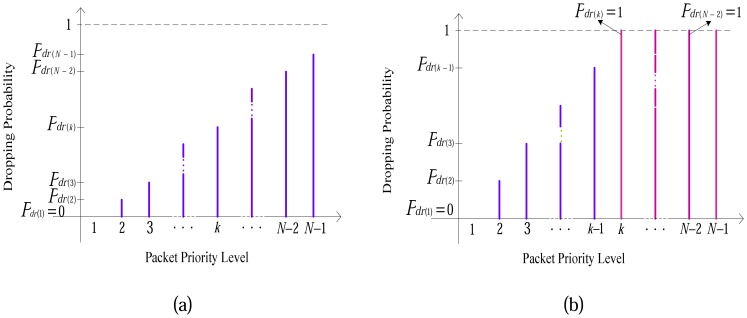
a) Dropping probability for packets with different priorities when energy state of node is higher than *ES_(N-1)_*, b) Dropping probability for the packets with different priorities in a node with energy state lower than *ES_(k)_*.

Furthermore, the values of dropping probabilities and energy state of the node have negative correlation in a way that when battery level decreases, dropping probability increases. When the energy level of a node is more than *ES_(N-1)_* (node has enough energy) the packet is selected with probability as shown in Figure 14.a. When the energy decreases to less than *ES_(k)_* the dropping probability increases as shown in Figure 14.b. The selection probability is constant between two consequent normalized energy levels and changes when it passes an energy threshold level (as has been shown in Figure 12).

The packet selection probability function can be defined with regards to application requirements. The function used for probabilities calculation should have a domain based on priority levels that node is allowed to send, and range between *0* and *1*. Also, it should be strictly increasing. The suggested function is:

(6) $$P_{dr(l)}=\{\begin{array}{ll} \frac{1}{P_{k}-1}\times (l-1)\times \beta & \;if\;0<l<P_{k}\\ \begin{array}{cc} 1 & \;\text{Otherwise} \end{array} & \end{array}$$

In this equation, *P_dr(l)_* is the dropping probability for packet with priority level (*l*), *P_k_* is the maximum priority level that VSN can transmit see Seccion 6.2.1, *l* is the priority level of current packet, and *β* is a coefficient which improves the flexibility of the Equation (6) which is defined between [0, 1]. After specifying dropping probability (*P_dr_*) for each packet, a random number *R* selected from [0, 1], if *R*<= *P_dr(l)_* packet is eligible for dropping, otherwise the packet is transmitted.

##### B. Hierarchical level based packet selection

In an energy level based scheme, the probability of discarding a packet is independent from the number of nodes that packet passed. Dropping a packet that is close to Sink (passed a lot of nodes) wastes more energy than dropping a packet close to the source node (only a few nodes passed) does. Thus, it is unfair to drop packets only on the basis of node energy. Consequently, an efficient packet discarding policy should consider preceding invested energy of each node. To achieve same drop probability for all packets (with the same priority level) received in the Sink, which originated from any node of the network, a fair drop selection policy should be defined. This motivates to find another probability factor to modify energy based drop probability. Therefore, the following equations should be satisfied:

(7) $$\begin{array}{l} P_{l11}^{\left(1\right)}=(1-\lambda )\\ P_{k22}^{\left(2\right)}=P_{k22}^{\left(1\right)}*P_{k12}^{\left(1\right)}=(1-\lambda )\\ P_{k33}^{\left(3\right)}=P_{k33}^{\left(1\right)}*P_{k23}^{\left(1\right)}*P_{k13}^{\left(1\right)}=(1-\lambda )\\ \;\vdots \\ P_{\text{kab}}^{\left(l\right)}=P_{\text{kab}}^{\left(1\right)}*P_{k(a-1)b}^{\left(1\right)}*\ldots *P_{k(a-(l-1))b}^{\left(1\right)}=(1-\lambda ) \end{array}$$

In these equations, $P_{\text{kab}}^{\left(l\right)}$ is the probability of receiving a packet in the base-station (Sink), where *l* is the number of levels that a packet should pass, *k* is packet priority level, *a* is the relying video node level, *b* is the source video node level, and *λ* is the constant that shows the specified dropping probability. It is optimistic to have functions that generate the related probabilities in the mentioned equations. Therefore, a close function is defined to calculate a probability that in conjunction with energy based packet drop probability results in a reasonable fair drop probability that somehow satisfies Equation (7). The proposed four-variable function is presented in Equation (8).

(8) $$F(a,b,t,n)=1-\{\begin{array}{lll} \frac{{\left[s-(a-t)\right]}^{n}}{{\left[s-(a-t)\right]}^{n}+{\left[t\right]}^{n}} & ,(s=b-a) & \;if[s-(a-t)]>=0\\ \begin{array}{ll} 0 & \;\text{Otherwise} \end{array} & & \end{array}$$

The presented function gives the second drop probability of each packet. In this equation *t* and *n* are two positive constant values that should be specified with regards to application. Both *a* and *b* are related to hierarchical levels (see Section 6.1. above) of relay and video capture node respectively.

Using this probability function along with energy based dropping probability, increases the receiving chance of the packets which passed more nodes than others. In other words, the receiving chance of packets generated by nodes close to the Sink is somehow the same as packets generated by nodes far from the Sink.

**Figure 15.**
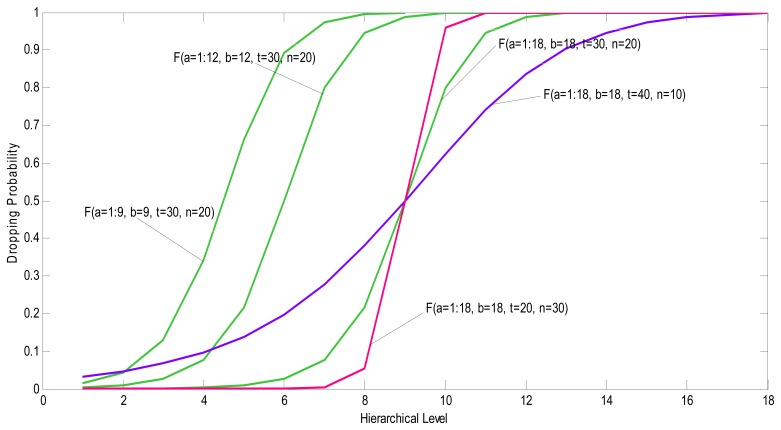
Effect of the different values on the proposed four-variable function in the Equation (8).

To drop a packet, a random number *R* ∈ [0, 1] is chosen. In case of *R* =< *F (a, b, t, n)*, the received packet will be dropped if it has energy based dropping condition, otherwise the packet is forwarded. Figure 15, illustrates the effect of some parameters on the function *F*(*a*,*b*,*t*,*n*) at a network with *18* hierarchical levels.

## 7. Simulation

The performance of proposed architecture was evaluated by MATLAB using Communication Toolbox and Video and Image Processing Block-set. Also, the required functions are generated by M-Files. The hierarchical network topology had *18* levels and *1000* video sensor nodes. All sensor nodes are considered homogeneous and their energy is supplied with two *1.5* V batteries. The video resolution is *320×240*. To perform the simulation it is assumed that all nodes have full battery, i.e., more than *ES(N-1)*. The parameter *∂* in algorithm 3 is set to *0.5* and the retransmission threshold value is *3*. Also in Equation 5 the parameter *z* is set to *2*, in Equation 6 the coefficient *β* is *0.5* and in Equation 8 the parameters *t* and *n* are set to *30* and *20*, respectively.

Moreover, Energy-Efficient and High Throughput MAC Protocol for Wireless Sensor Networks (ET-MAC) [40] is used in data link layer for simulations. In order to get precise results from the simulations the parameters of CC2420 [41] Chipcon transceiver listed in Table 1, were used.

### 7.1. Selecting Quality Coefficient

The *quality coefficient* parameter affects the value of each element in quantization luminance matrix [34]. As mentioned in Section 4.1, an M-Frame block is prepared for transmission by dividing blocked DCT matrix with the quantization luminance matrix. Hence, *quality* influences the value of each pixel in M-Frame blocks. Choosing an appropriate value for *quality coefficient* is so important, since small value for it leads to a greater number of bits required for transmitting the data of each block pixel. On the other hand, high value for *quality* results in a greater number of zeros in each M-Frame block. This means more data loss in M-Frame compression method. As a result, defining *quality coefficient* encounters the bit-loss trade off.

**Figure 16.**
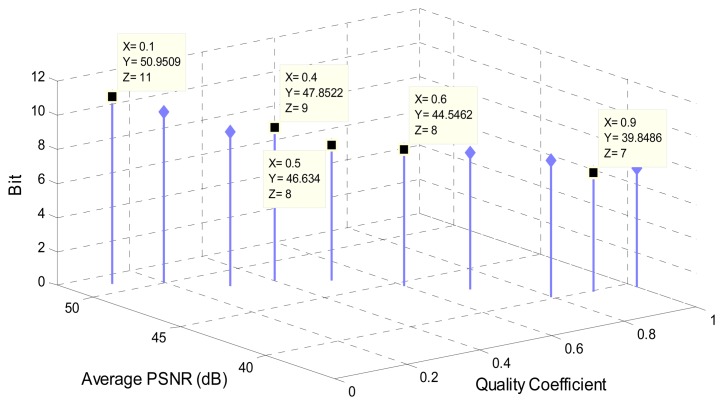
The relation between *quality coefficients*, average PSNRs, and number of bits per pixel.

The approach used for defining *quality* is examining different values for it. By analyzing average Peak Signal-to-Noise Ratio (PSNR) [42] and required bit in different *qualities* in various video frames, it is shown that some *quality* values require *8* bits for transmitting each pixel of M-frame blocks. Since *8* bits are earmarked for each pixel, the value with negligible loss (proper PSNR) is selected. As Figure 16 shows, *0.5* is the best value for bit-loss trade off which also used in our simulations.

### 7.2. Selecting Maximum Priority Level

The parameter *N* is the number of priority levels which are selected for transmission. Each one of the mentioned priority levels includes some pixels of a frame. High values for *N* result in forwarding a large number of pixels for M-Frame blocks. This causes increase in energy consumption and bandwidth usage, as well as improvement in video quality. On the other hand, transmitting few priority levels has opposite consequences. Comparing the average PSNR and pixels for all values of *N* from *1* to *13*, we concluded that *7* is an acceptable value for *N* and this value is used in simulations. Table 2, contains these results.

### 7.3. Analyzing Energy Efficiency and Video Quality for Different Environments

In this section, we evaluated the performance of presented architecture. The behaviors of designed architecture should be considered with the aim of having actual results for our simulation. As mentioned in Section 4.3, the period of forwarding M-Frames depends on the number of the pixels which are used for delivering M-Frames and D-frames. Moreover, priorities assigned to D-Frame blocks are related to the difference between pixels of current D-Frame and saved previous M-Frames. Thus, the performance of the architecture is associated with the variation degree of the environment. Owing to this fact, examined environments are divided into four categories: still environment, little-variant environment, middle-variant environment, and high-variant environment. The performance is calculated in each of these categories.

**Figure 17.**
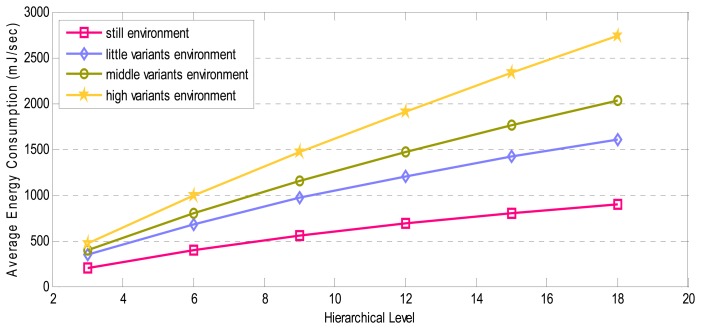
Average Energy consumption for the entire network in different environments

In the following, the average result of five video samples captured by video nodes located in hierarchical levels from *3* to *18* during *10* seconds in each environment is presented. Figure 17, shows the relation between the energy consumed and hierarchical levels for tested environments. Having analyzed simulation results, we concluded that energy consumption depends on the variants in the examined environment. When variation degree of the environment increases, M-Frames are forwarded with higher frequency because of more different pixels between captured frame and saved M-Frame. This leads to more pixels of D-Frames to be sent. Consequently, the average energy consumption increases and high-variant environments consume more energy in comparison with other environments (as shown in Figure 17).

Also, the level of the source node in the hierarchical topology influences the energy consumption of the whole network. Since the location of the source node determines the number of contributed intermediate nodes in delivering video frames, energy consumption increases when number of relaying nodes grows.

**Figure 18.**
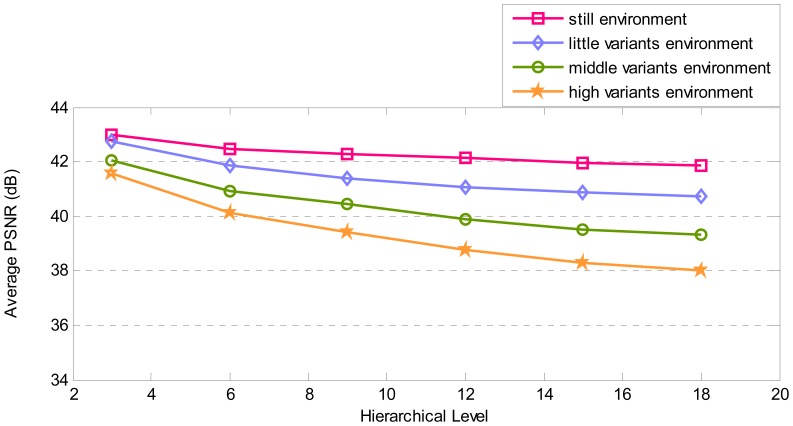
Average PSNR for different environments

Another factor to be analyzed is video quality which is inspected by average PSNR. Figure 18, elucidates the correlation between average PSNR and hierarchy level. Average PSNR changes in associated with variants in the environment and hierarchy level of video source node like consumed energy. The major reason of this fact is the impacts that dropping scheme has on average PSNR. In high-variant environment, more pixels change; thus, sending more pixels for a frame is needed. Therefore, on the same probability of packet dropping, more pixels of the frame are dropped through transmission and smaller number of frame's pixels is received in Sink. As a result, average PSNR is lesser than other categories of environments. Interpretation of the result of simulation in EQV-Architecture shows satisfactory functionality.

**Figure 19.**
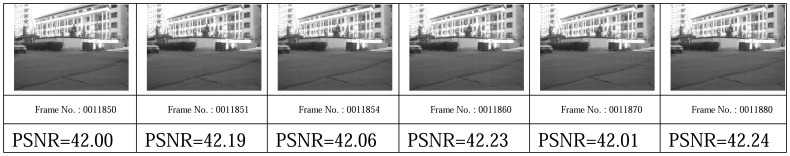
Still environment frames

**Figure 20.**
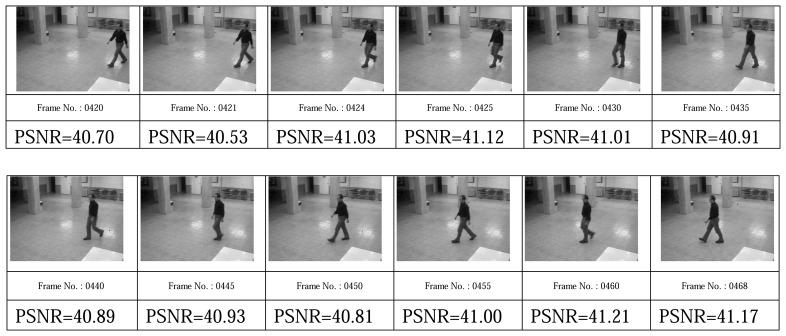
Little-variation environment Frames

**Figure 21.**
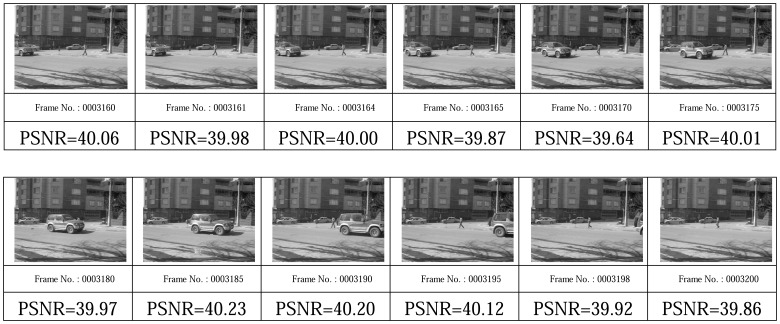
Middle-variation environment frames

**Figure 22.**
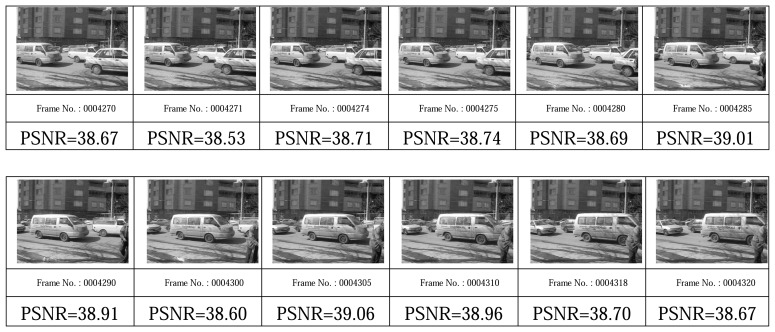
High-variation environment frames

Figures 19, 20, 21, and 22 show some video frames from different categories of environments with their PSNRs in receiver side. These frames are samples that were used to calculate average PSNRs in Figure18.

### 7.4. Effect of Random Early Dropping Method on Energy and Video Quality

Random early dropping is presented in Section 6.2.2 as an approach for extending the lifetime of the WVSN. Although this scheme has various benefits, it influences the video quality. In this section, two criteria are utilized for evaluating the performance of dropping scheme. The criteria are average bytes which are forwarded from source to Sink and average PSNR in receiver side. Video is transmitted first by using dropping scheme and then without using this scheme. It is assumed that all nodes have full batteries and the environment examined is a mixture of all four categories of environments. Furthermore, simulation lasted for *10* seconds.

The simulation results shown in Table 3, are evidence that the number of sent bytes in dropping scheme decreases. According to Equation (9), the optimization of the sent bytes in the dropping scheme is *24%*.

(9) $$\text{Performance}=\frac{\sum_{i=3}^{18}{\left(\text{Average transmitted bytes without dropping}\right)}_{i}-\sum_{i=3}^{18}{\left(\text{Average transmitted bytes with dropping}\right)}_{i}}{\sum_{i=3}^{18}{\left(\text{Average transmitted bytes without dropping}\right)}_{i}}$$

In this equation, *i* is the hierarchical level that video capture node is situated on.

**Figure 23.**
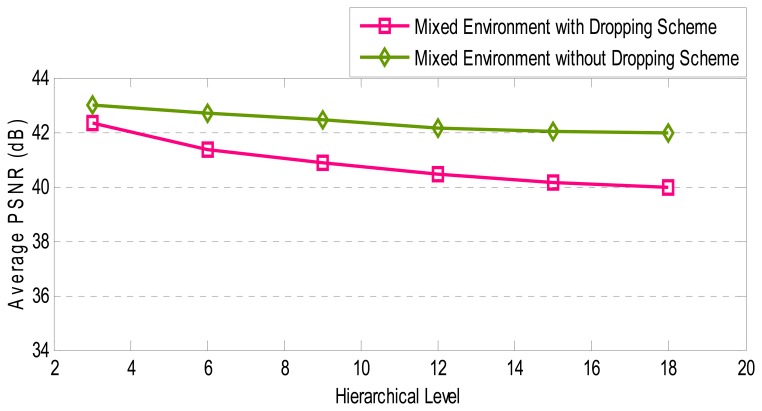
The average PSNR for Mixed Environment with and without Dropping Scheme

On the other hand, Figure 23 shows that average PSNR is reduced by approximately *1.51* dB. The resulted value, however, is a satisfactory average PSNR for video transmission. Based on the achieved results, it is reasonable to use dropping method for achieving energy efficiency. Dropping scheme takes account of energy and hierarchical levels of nodes and data importance for dropping. Therefore, it drops low-priority data while having in view the level of source node. In short, average PSNR does not change a lot even when about one quarter of the data is dropped.

### 7.5 Comparing EQV-Architecture with Other Protocols

In order to evaluate the EQV-Architecture effectively, it is compared with another architecture composed of three different protocols. In the set of selected protocols, there were protocols compatible with real-time transmission and multi-path routing. The chosen protocols were: MPEG-2 in application layer, MRTP [24] in transport layer, and MMSPEED [31] in network layer. The simulation performed in 5 sample of mixed environment.

**Figure 25.**
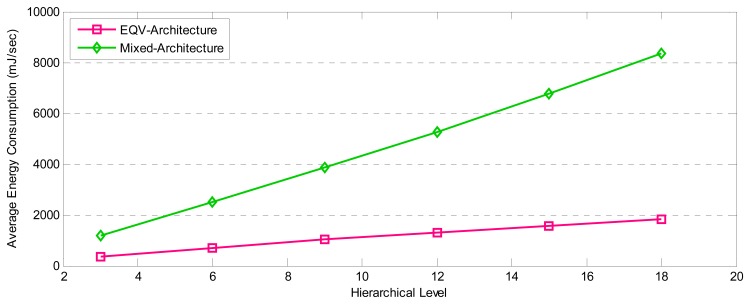
The comparison of average energy consumption between EQV-Architecture and Mixed-Architecture

Figure 25, shows the comparison of average energy consumption in these two architectures. EQV-Architecture saved more energy than Mixed-Architecture does. That is due to the fact that Mixed-Architecture sends I-Frames periodically [34] and delivers packets in multi-paths, while EQV-Architecture utilizes dynamic period for transmitting M-Frames in single-path hand in hand with dropping scheme. Therefore, EQV-Architecture saves approximately *75%* of energy.

**Figure 26.**
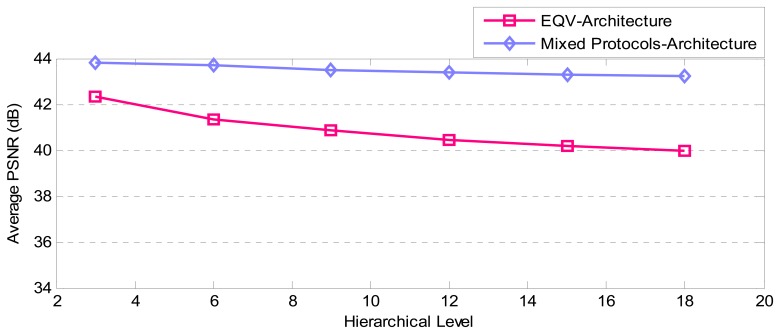
The comparison of average PSNR between EQV and Mixed Architectures

Another point for comparison is video quality based on average PSNR which is illustrated in Figure 26. It shows that however EQV-Architecture provides less PSNR than Mixed-Architecture, the resulted PSNRs are satisfactory.

## 8. Conclusions

In this article the EQV-Architecture for video transmission in wireless multimedia sensor networks is presented. Battery awareness and video quality issues are considered in application, transport and network layers of communication protocol stack. A prioritized video compression protocol in application layer and new transport layer protocol along with two dropping schemes and single-path routing protocol in network layer are introduced in this architecture. The algorithms, methods, and protocols presented here are in accordance with WVSN and support two mentioned issues.

Simulation for presented architecture is applied in various environments with different variants from the perspectives of both energy-efficiency and video quality. Simulation results indicate that the optimization of energy consumption and also video quality in proposed architecture differ in disparate environments, but this architecture has better performance than other conventional architectures. In other words: EQV-Architecture extend the lifetime of the networks while providing sufficient video quality. In the future, architecture with ability to correspond multi-video requests from Multi-Sinks will be investigated.

## Figures and Tables

**Table 1.** CC2420 transceiver parameters

**Parameter**	**Value**
Bit Rate	250 k_bps_
Listen Power	60 mJ/sec
Receive Power	63 mJ/sec
Transmission Power	57 mJ/sec
Setup Time	1msec
Communication Distance	300m

**Table 2.** Corresponded Average PSNR and Number of Transmitted Pixels for different value of *N*

*N*	2	3	4	5	6	7	8	9	10	11	12	13
Average PSNR	30.79	33.93	36.58	39.00	41.58	44.30	45.44	45.80	46.23	46.31	46.34	46.35
Number of Transmitted Pixels	3	6	10	15	21	28	36	43	49	54	58	61

**Table 3.** The average Transmitted Data in transmission with Dropping Scheme and without it

Hierarchical Level	3	6	9	12	15	18
Transmitted Data with Dropping Scheme (MB)	39.12	79.51	114.85	146.54	175.45	202.21
Transmitted Data without Dropping Scheme (MB)	44.88	95.99	144.50	190.72	234.88	277.17
